# Enablers and barriers to the secondary use of health data in Europe: general data protection regulation perspective

**DOI:** 10.1186/s13690-022-00866-7

**Published:** 2022-04-09

**Authors:** Jakov Vukovic, Damir Ivankovic, Claudia Habl, Jelena Dimnjakovic

**Affiliations:** 1 Division for Health Informatics and Biostatistics, Croatian Institute of Public Health, Rockefellerova, Street 7, 10 000 Zagreb, Croatia; 2 Academisch Medisch Centrum Universiteit Van Amsterdam, Meibergdreef 9, 1105 Amsterdam, AZ Netherlands; 3 Austrian National Public Health Institute, Stubenring 6, 1010 Vienna, Austria

**Keywords:** GDPRGeneral Data Protection Regulation, Secondary health data, Data sharing

## Abstract

**Background:**

The General Data Protection Regulation is a regulation in EU law on data protection and privacy in the European Union. We aimed to provide an overview of the General Data Protection Regulation (GDPR) enablers and barriers to the secondary use of health data in Europe from the research we conducted in the Joint Action InfAct (Information for Action!) WP10 Assessing and piloting interoperability for public health policy, as well as to provide an example of a national-level case study on experiences with secondary use of health data and GDPR on an example of the Austrian COVID-19 data platform.

**Methods:**

We have identified a number of European initiatives, projects and organizations that have dealt with cross-border health data sharing, linkage and management by desk research and we conducted 17 semi-structured in-depth interviews and analyzed the interview transcripts by framework analysis.

**Results:**

GDPR was seen as an enabler to the secondary use of health data in Europe when it comes to user rights over their data, pre-existing laws regarding data privacy and data sharing, sharing anonymized statistics, developing new data analysis approaches, patients` trust towards dealing with their health data and transparency. GDPR was seen as a barrier to the secondary use of health data in Europe when it comes to identifiable and individual-level data, data sharing, time needed to complete the process, workload increase, differences with local legal legislations, different (and stricter) interpretations and access to data.

**Conclusion:**

The results of our analysis show that GDPR acts as both an enabler and a barrier for the secondary use of health data in Europe. More research is needed to better understand the effects of GDPR on the secondary use of health data which can serve as a basis for future changes in the regulation.

## Background

The General Data Protection Regulation (GDPR) is a regulation in EU (European Union) law on data protection and privacy in the European Union [1]. The objective of the GDPR is to facilitate the free movement of personal data and cross-border data exchange, as well as to protect the fundamental rights and freedoms of European citizens [2]. It came into effect in 2016 and became applicable in all EU Member States on May 25, 2018 [2]. It replaced the 1995 Data Protection Directive [3] which was adopted prior to the mainstream adoption of the internet. Secondary use of health data refers to the usage of aggregated health data from sources such as electronic health records, health insurance claim data and health registry data.

We aimed to provide an overview of the GDPR enablers and barriers to the secondary use of health data in Europe from the research we conducted in the Joint Action InfAct (Information for Action!) on Health Information co-funded by the Third EU Health Programme (2014–2020) which includes 40 partners in 28 countries with the goal of strengthening national and EU health information systems by establishing a sustainable EU health information system infrastructure. Our research was done as a part of the work package titled 10 “Assessing and piloting interoperability for public health policy” which dealt with mapping and analysing national and international inspirational cases with the focus on public health interoperability and developing empirical work on interoperability through case studies and pilots with a interoperability of re-usable and “actionable” data.

The main objective of this work is to provide insights into the relation between a regulation – GDPR—and data practice – secondary use of health data. It does so by specifically exploring how reearchers working on cross-border projects and exchanging health data…see the GDPR, how it affects their work, where they see the GDPR as an enabler and where as a barrier to the cross-border health data exchange. In addition, to illustrate this, the article presents a country-specific case study from Austria on experiences with secondary use of health data and GDPR along the lines of the national COVID-19 data platform that provides data to national and international researchers.

## Methods

We aim to provide an overview of the GDPR enablers and barriers to the secondary use of health data in Europe and present a country-specific case study on the topic.

In order to achieve these goals this work was conducted in two steps: 1) Identification of GDPR-related enablers and barriers to cross-country health data exchange in Europe from a legal perspective (further: legal-level enablers and barriers) and 2) country specific case study validating and contextualizing some of the findings with the example of the Austrian COVID-19 data platform.. All work was done during the EU Joint Action on Health Information (www.inf-act.eu).

### GDPR-related enablers and barriers to cross-country health data exchange in Europe

We have conducted semi-structured in-depth interviews with participants from European initiatives, projects and organizations that have dealt with cross-border health data sharing, linkage and management to find out what are the legal, GDPR-related enablers and barriers to cross-border health data exchange in Europe and how are European countries dealing with secondary use of health data since the implementation of the GDPR.

During the European Joint Action Infact we identified a number of European initiatives, projects and organizations that have dealt with cross-border health data sharing, linkage and management, leading to 17 personal, structured interviews with leading experts in population and digital health in Europe.

The interview instrument was developed with a specific aim of being used for semi-structured interviewing techniques involving InfAct researchers, as interviewers, and representatives of cross-country health data exchange projects in scope, as interviewees.

European Interoperability Framework (EIF) layers of interoperability have been used as a starting point for the development and structuring of the interview instrument. Meant as a list of questions for a semi-structured interview, the instrument consisted of a number of sections. It started by asking interviewees general questions about the project / initiative and continued with questions related to four interoperability layers (legal, organizational, semantic and technical). In this paper we discuss only the GDPR specific questions related to the legal layer of interoperability.

Interviews were conducted either using online teleconferencing software (Skype and GoToMeeting) or through in-person meetings. 15 interviews were conducted in English and two in Croatian. With participants’ consent, interviews were recorded. Researchers transcribed the recordings and qualitatively analyzed respondents’ answers.

Interviews were conducted by three InfAct researchers from the Croatian Institute of Public Health. For the qualitative thematic analysis, we used NVivo 12 Pro software. After multiple readings of interviews, we constructed the coding scheme that largely resembles the structure of our questionnaire.

In the period between July 2019 and April 2020, we conducted a series of 17 interviews based in a number of different European countries. Through these semi-structured interviews our main goal was to discuss in-depth how these projects and initiatives tackled issues related to data sharing, linkage and management as well as to learn about the enablers and barriers in achieving project goals. We did so by steering the discussion through the four interoperability layers, as described above, leaving ample time and space for discussions on specifics of day-to-day operation of cross-border health data exchange in the discussed projects.

Interviews had a “mixed profile” of invitees, both top-level project coordinators as well as national-level partners in order to capture a wide range of issues presented as both enablers and barriers on all levels of dealing with projects. Also, by not excluding national-level participants, we are able to get more information on the national implications and use, for policy- and decision-making, of the results from these projects.

Following the finalization of all interviews, recordings were transcribed and the transcripts of all interviews were analyzed by framework analysis using NVivo software. Transcripts were coded using line by line coding and the codes were grouped into four layers of interoperability (legal, semantic, organisational and technical). These were further grouped into enablers and barriers for each layer of interoperability based on the interpretation of the codes. In this paper we are going to focus on the legal layer of interoperability with the focus on our results on the GDPR enablers and barriers.

### National-level case study on experiences with secondary use of health data and GDPR—Example of the Austrian COVID-19 data platform

Experience with secondary use of health data and GDPR is illustrated with the establishment of the national COVID-19 data platform (https://datenplattform-covid.goeg.at/english). The data platform was designed and developed by the Austrian National Public Health Institute Gesundheit Österreich GmbH (GÖG) on request of the Austrian Minister of Health in an early stage of the COVID-19 pandemic in spring 2020. Originally intended to share data in a pseudonymised way this had to be replaced by data sharing only in an anonymized way as the national data protection rules did not allow for the first.

## Results

### GDPR-related enablers and barriers to cross-country health data exchange in Europe

Our interview tool was constructed to discuss all four layers of interoperability (legal, technical, organizational and semantical). Discussion on legal issues made up one fifth of the themes covered during the interviews with the discussion about GDPR being close to half of the discussion on the legal layer of interoperability. GDPR enablers made up to one tenth and GDPR barriers made up to one quarter of the discussion on the legal layer.

**Fig. 1:**
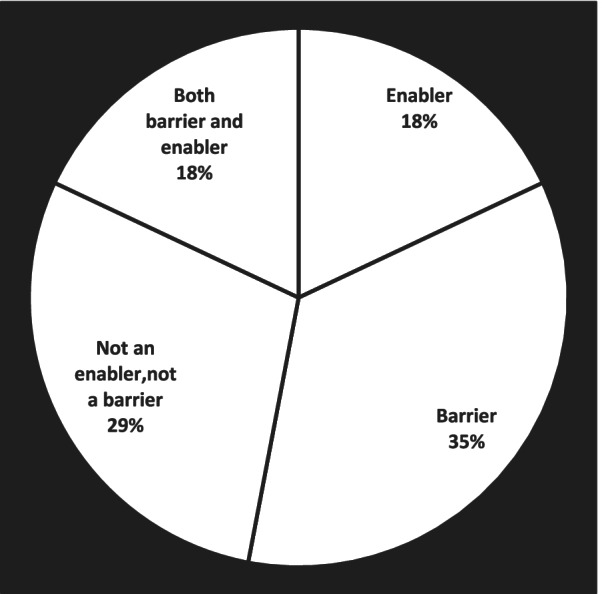
Sentiment towards the General Data Protection Regulation (GDPR) and its effect on cross-border health data sharing, linking and managing

The sentiment towards the General Data Protection Regulation (GDPR) and its effect on the cross-border health data sharing, linking and managing showed that third of the participants perceived GDPR as a barrier for secondary use, one fifth as an enabler, one fifth both as an enabler and a barrier, and one third considered it neither as a barrier nor as an enabler (Fig.  1).

GDPR was seen as an enabler for the cross-border health data sharing, linking and managing when it comes to the user rights over their own data as it helps with defining the user ownership and right over the data and specific purposes for which the data can be used. Also, GDPR facilitates development of tools giving more control over the data to data owners (patients) and data custodians (organizations) which was seen as a significant feature of the GDPR as both patients and organizations today are becoming more and more sensitive and aware of the importance of the ownership and usage of the own personal data.

GDPR implementation was easier in countries with pre-existing legislation on data privacy and data sharing regulation. This made complying with the new GDPR regulation easier, while the countries which did not have pre-existing laws regarding data privacy and data sharing had to implement and enforce the new regulation.

When it comes to anonymized statistics, GDPR is seen as an enabler as it actively defines, enables and encourages sharing of anonymized statistics. This can be attributed to the fact that the GDPR makes sharing identifiable data a bit more difficult, while it does not pose any obstacles towards sharing anonymized data. There are much more concerns about data protection, partly due to implementation of the GDPR, which makes it more difficult to share data for scientific purposes. GDPR limits some projects to only share the aggregated data as a way to avoid sharing individual-evel data and the GDPR challenges that come with that. 3^rd^ parties are unable to access to individual data, only aggregated results are shared with them. Informats reported that GDPR and privacy concerns are sometimes used as an excuse to stop sharing the data when people are in fact not willing to share the data. Data providers are concerned about eventual violation of the data protection laws, which leads some countries to stop sharing their data.

The implementation of GDPR was a facilitator for the introduction of new data analysis approaches which do not require full data sets to be shared as it minimizes data privacy risks, such as federated data exchange and management. Informats reported that this can be attributed to the human nature of problem solving as the implementation of the new regulation meant that some of the old ways of analyzing the data were not complying with the new regulation and new approaches were developed to adapt to the new regulation and continue the data analysis and data sharing while at the same time minimizing data privacy risks. When developing novel approaches to dealing with health data, solutions tend to be restrictive to ensure the compliance with GDPR in all the countries.

One of the biggest reported benefits of the GDPR was the positive effect on patients` trust towards dealing with their health data as it makes the system safer and helps with keeping the trust of patients when it comes to dealing with their health data, as well as the transparency which GDPR enables, which in turn facilitates the relationships between the stakeholders as the agreements between them are more transparent which is beneficial for research.

GDPR was seen as a barrier when it comes to identifiable and individual-level data as it represents an issue when dealing with health data narrowly defined by region, sex, age group and International Classification of Disease (ICD) code where the size of the sample is very small (1, 2 or 3 persons) as it could be a way of identifying individuals. This is especially seen as a barrier when it comes to the rare events where the data is potentially identifiable. When dealing with individual-level data, GDPR was a great concern as everything is potentially confidential and re-identifiable and makes working with anything resembling individual-level data complicated as everything is potentially confidential and identifiable.

Countries interpret GDPR in a softer or stricter way and there are differences amongst member states in the concrete implementation of the GDPR into national regulations. Sometimes the national implementation seems to be even contradictory to the GDPR. There are interpretations of the GDPR which are stricter than it was intended with the GDPR and a lot of people over-interpret the GDPR and make it stricter than it was intended. There is a contradiction in the interpretation of the GDPR between reading it word by word and the spirit and the purpose of the GDPR, which makes the interpretation of the GDPR stricter to ensure compliance with it. There are different interpretations of the GDPR which represents a barrier. GDPR is a unique and interesting regulation but the interpretation and implementation of the GDPR has caused problems and represents a challenge in Europe which needs to be addressed.

While the idea of the GDPR was not to make research more difficult, it sometimes slows down the process. While the same research can still be conducted, the process to comply with GDPR slows down the project and complicates it. Implementing GDPR represents a major work burden in projects which deal with cross-border health data exchange as they have limited budgets from research funding and limited personnel and the legal issues take much more time and work than it is available which restricts carrying out the project simultaneously while and there is a lack of funding to set up data and information exchange systems which would be compliant with the GDPR. Therefore, the workload to be GDPR compliant is a barrier for projects.

Table 1 Presents areas where General Data Protection Regulation (GDPR) was seen as an enabler for cross-border health data sharing, linking and managing by our interview participants.

**Table 1:** General Data Protection Regulation (GDPR) as an enabler for cross-border health data sharing, linking and managing

GDPR as an enabler	
Pre-existing laws regarding data privacy and data sharing	GDPR is easier to implement in countries with previously existing and implemented laws regarding data privacy and data sharing
Anonymized statistics	GDPR defines and enables sharing of anonymized statistics
New data analysis approaches	GDPR is a facilitator for the introduction of new data analysis approaches which do not require full data sets to be shared as it minimizes data privacy risk
Patients` trust towards dealing with their health data	GDPR is a big asset for Europe as it makes the system safer and helps with keeping the trust of patients when it comes to dealing with their health data
Transparency	GDPR enables transparency and facilitates the relationships between the stakeholders GDPR is an enabler as having more transparent agreements can only be beneficial for research and for relationships of all the stakeholders
User rights over their data	GDPR helps with defining the user ownership and right over the data and specific purposes for which the data can be used It also facilitates development of tools which give control over the data to data owners (patients) and data custodians (organizations)

Table 2 Presents areas where GDPR could be considered as a barrier for cross-border health data sharing, linking and managing by our interview participants.

**Table 2:** General Data Protection Regulation (GDPR) as a barrier for cross-border health data sharing, linking and managing

GDPR as barrier	
Data sharing	There are much more concerns about data protection which makes it more difficult to share data for scientific purposes GDPR limits some projects to only share the aggregated data as a way to avoid sharing individual-level data and the GDPR challenges that come with that
GDPR implementation	GDPR is a unique and interesting regulation but the interpretation and implementation of the GDPR has caused problems and represents a challenge in Europe, which needs to be addressed
Time	GDPR slows down the process. The idea behind GDPR is not to make research more difficult, the same research can still be conducted but the process is just slower and more complicated
Workload (and resources) involved in GDPR compliance	Implementing GDPR is a major work burden and represents a problem in projects, which work with limited budgets from research funding and limited personnel, as the legal issues take much more time and work than it is available which restricts carrying out the project simultaneously The workload to be GDPR compliant is a barrier for projects There is a lack of funding to set up data and information exchange systems, which would be compliant with the GDPR
Local legislation	There are differences in national interpretation and implementation between countries; and sometimes national regulations are contradictory to the GDPR
Different (and stricter) interpretations	Locally there are differences between countries as to how strict they are about the interpretation of the GDPR and specific laws, which represent a barrier There are interpretations of the GDPR, which are stricter than it was intended with the GDPR A lot of people over interpret the GDPR and make it stricter than it was intended
GDPR implementation in countries without pre-existing laws concerning data privacy	GDPR did not make a big difference in countries with an already strict legislation, while it did have an impact on countries where a strict legislation did not exist prior to the implementation of the GDPR
Access to data	Access to individual data is restricted to 3rd parties, only aggregated results are shared GDPR and privacy concerns are sometimes used as an excuse to stop sharing the data Data providers are concerned about eventual violation of the data protection laws, which leads some countries to stop sharing their data
GDPR interpretation	There is a contradiction in the interpretation of the GDPR between reading it word by word and the spirit and the purpose of the GDPR Lawyers are not sure how to interpret GDPR, which, in the end, makes the interpretation of the GDPR stricter to ensure compliance with it. There are different interpretations of the GDPR, which represents a barrier
Novel approaches towards health data	When developing novel approaches to dealing with health data, solutions tend to be restrictive to ensure compliance in all the countries
Identifiable and individual-level data	GDPR is an issue with health data narrowly defined by region, sex, age group and International Classification of Disease (ICD) code where the size of the sample is very small (1, 2 or 3 persons) as it could be a way of identifying individuals When it comes to rare diseases, data is potentially identifiable. There is a great concern when dealing with individual level data as everything is potentially confidential and re-identifiable GDPR makes it complicated to work with anything resembling individual-level data as everything is potentially confidential and identifiable

### National case study on experiences with secondary use of health data and GDPR—Example of the Austrian COVID-19 data platform

The availability of health and care data for research in Austria is traditionally limited due to ownership and data protection issues. With the outbreak of the COVID-19 pandemic the Austrian research community heavily demanded access to data and information (e.g., use of resources, patient pathways)—not only for scientific purposes but also to aid in risk-assessment and crisis management. As the requested information (e.g., use of resources, patient pathways, ICU capacities) involved personal and health care data it became necessary to establish strict rules for this information provision.

**Fig. 2:**
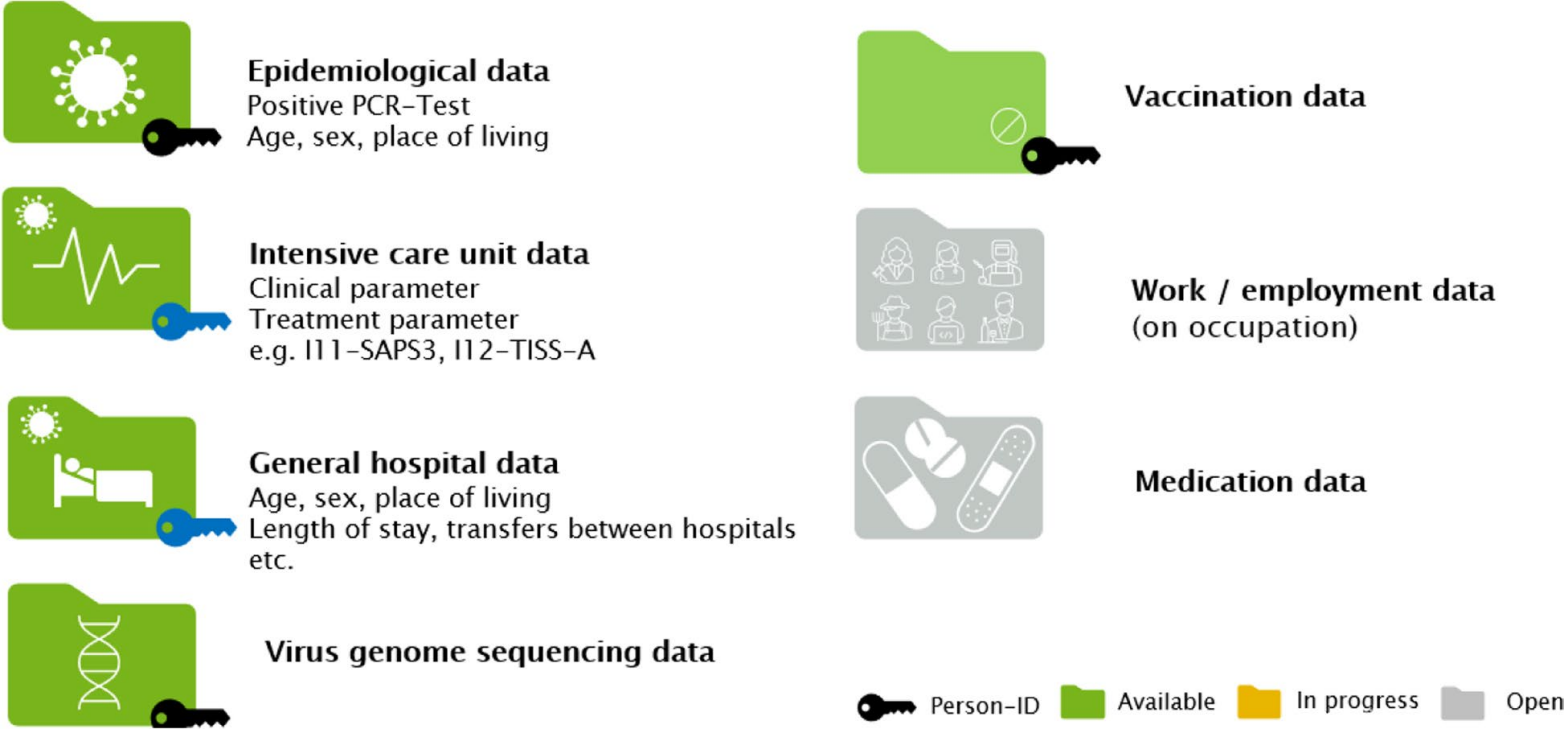
Graphical presentation of data contained in Austrian platform

In the absence of both, a national data ethics committee or a data clearing house the owner of the data, the Austrian Ministry of Social Affairs, Health, Case and Consumer protection (BMSGPK) commissioned the Austrian National Public Health institute Gesundheit Österreich GmbH (GÖG) to establish a GDPR compliant tool to provide this information. Within a couple of weeks GÖG set up the national COVID-19 data platform (https://datenplattform-covid.goeg.at) and the necessary governance structure. The latter include a comprehensive and transparent application process under the auspices of a scientific advisory board of researchers, including top national data protection experts. The data provision started with information from the epidemiological reporting system and was complemented over time by information on regional data, hospital stays (diagnosis and treatment), genome sequencing data and since December 2021 vaccination data (Fig. 2).

To make information provision to researchers possible, a number of the above-mentioned barriers, namely (physical) access, national (strict) interpretation of existing laws as well as the resources and time had to be overcome. A core activity was the modification of the national Epidemics Act (Epidemiegesetz 1950) by adding Art. 4a [5] enabling GÖG to provide the information for researchers [4].

Another core enabler was the decision to use k-anonymity (concept for the protection of privacy developed by L. Sweeny in 2002 to protect the health data of patients [5]. Amongst other the following measures were taken:

- Persons < 20 years and > 79 years were each merged into one group;
- Remaining persons were clustered in age groups spanning 15 years;
- No information is shared on community level unless at least patients with the same post code were identified in the data set;
- Nationality of patients is not included in the standard data set (but can be added if requested and if the scientific advisory board agrees);
- Hospital stays are counted in weeks, not days and detailed billing information is not provided.

Data included in the platform are linkable to each other, but no linkage to other registries is currently possible. A change would require several legal changes, namely a regulation based on the Austrian Research Organisations Act (Forschungsorganisationsgesetz) [6].

When setting up the platform other activities in Europe in this field were quickly analysed, to identify good practices e.g. like the Finnish Social and Health Data Permit Authority (Findata) [7].

In the meantime, the COVID-19 platform could already support more than 100 requests for information leading in several scientific presentations. Some of the data use requests came from other countries, despite it was neither broadly announced nor marketed. This is a clear indication for existing cross-country research and expert networks like the one that is intended with the planned EU Health Information portal (www.healthinformationportal.eu), which was the inspiration for this article.

The COVID-19 pandemic and the data platform triggered a national debate around the need for a national data governance framework balancing the benefits of the usage of personal health data for research and public health purposes versus the right to data protection of citizens’ health data.

This boosted previous attempts to provide more administrative data and routinely collected information for research purposes and led to the announcement of the Minister of Science in July 2021 to establish an ‘Austrian Micro Data Centre’ where selected data on education, employment, place of living and selected health care data shall be available for secondary use in summer 2022. To establish this Centre existing legislation needs to be amended, e.g., the Federal Statistics Law (Bundesstatistikgesetz) and the Research Organisations Act. The Austrian Federal Bureau for Statistics Austria will be in charge of implementation. Together with the implementation of GÖG as national node for population health (www.healtinformationportal.eu) this is an important step towards a European Health Data Space.

## Discussions

We conducted our interviews to find out what are the legal, GDPR-related enablers and barriers to cross-border health data exchange in Europe and how are European countries dealing with secondary use of health data since the implementation of the GDPR. We used a country-level case study to illustrate, validate and contextualize our findings on the example of the Austrian COVID-19 data platform.

While our interviews were designed to get an insight into all four layers of interoperability (legal, technical, semantical and organizational) when dealing with cross-border health data sharing, linking and managing, our semi-structured approach to the interviews gave the opportunity to the interview participants to stress out the areas which they believe are of most importance when it comes to the cross-border health data sharing, linking and managing. In our interviews we included participants from all levels of project hierarchy to gain a complete insight into possible GDPR-related enablers and barriers to cross-border health data exchange in Europe. Limitation of this approach was that all interview participants did not discuss the same issues as their experiences varied based on their place in project hierarchy and their own professional experience when dealing with cross-border health data exchange in Europe.

The results of our analysis show that GDPR acts as both an enabler and a barrier for the secondary use of health data in Europe. It is seen as an enabler as it enables user`s rights over their data, increases patients` trust towards sharing their data, enables sharing of the anonymized statistics, increases transparency between partners and facilitates the introduction of new data analysis approaches which do not require full data set to be shared which in turn minimizes data privacy risk. GDPR is seen as a barrier when it comes to dealing with identifiable and individual-level data and different GDPR interpretations and implementations in different countries, as well as an unclear understanding of it are seen as a barrier for cross-border health data exchange in Europe. The Austrian example showed that GDPR stipulation could be hard to fulfill in practice. Originally the COVID-19 data platform was intended to share data in a pseudonymised way, but this had to be replaced by data sharing in an anonymized way as the national data protection rules did not allow for the first.

Bankava noted that there is no common understanding of techniques and criteria used in order to anonymize identifiable personal data and situations can occur where data subjects can be identified [8] which is in line with our results as the GDPR was seen as a barrier when it comes to identifiable and individual-level data as it represents an issue when dealing with health data narrowly defined by region, sex, age group and ICD code where the size of the sample is very small (1, 2 or 3 persons) as it could be a way of identifying individuals.

While The European Data Protection Supervisor (EDPS) issued a Preliminary Opinion that there is no evidence that the GDPR itself hampers genuine scientific research [8], Soini noted that his own personal experience is different and he has witnessed insurmountable legal obstacles to share data globally [9] and Greene noted that data scientists and researchers are facing uncertainty and confusion regarding their routines and practices, as well as pressures from legal advisers [10] which is in line with the experiences of our interview participants as they emphasized that even though the same research can still be conducted, GDPR represents a major work burden in projects which deal with cross-border health data exchange as they have limited budget and limited personnel and the legal issues take a lot of time.

There are complex variations among EU member states when it comes to GDPR [11] and Negrouk noted that there is a poor and divergent interpretation of the GDPR, as well as a lack of guidance relevant to health research. Negrouk also noted that there are over-interpretations of the GDPR and increasing restrictions imposed on research have been justified as being GDPR compliant [12] which is in line with our results as our interview participants stressed out that locally there are differences between countries as to how strict they are about the interpretation of the GDPR, as well as differences in local regulations between countries which sometimes differ from GDPR. There are interpretations of the GDPR which are stricter than it was intended with the GDPR and a lot of people over-interpret the GDPR and make it stricter than it was intended. Clarke noted that The Irish Department of Health has taken a unique and arguably restrictive approach to data protection in Ireland which is quite at variance from other European approaches [13]. van Veen noted that different national implementation of the research exemptions may lead to problems in international research projects [14] which is in line with our results as our interview participants stressed it out as one of the main barriers for cross-border health data exchange projects. Pillips noted that careful attention is needed to the country-specific implementations and interpretations of key aspects of data protection [15] which was also emphasized in our interviews as a univocal interpretation of the GDPR might remove many of the restrictions that the data providers currently have.

National-level case study on the example of the Austrian COVID-19 data platform shows how Austria established a GDPR compliant tool to provide information (e.g., use of resources, patient pathways, ICU capacities) and which barriers had to be overcame to make information provision to research possible. The COVID-19 pandemic and the data platform triggered a national debate around the need for a national data governance framework balancing the benefits of the usage of personal health data for research and public health purposes versus the right to data protection of citizens’ health data.

During literature review we noticed that the other GDPR enablers and barriers for the secondary use of health data in Europe from our results are not addressed in scientific publications apart from the above-mentioned citations. Our results show some clear benefits from the GDPR for the secondary use of health data in Europe and we see a need for a deeper understanding of those benefits for the research community. On the other hand, our results show GDPR barriers for the secondary use of health data in Europe which should be addressed. Different interpretations of the GDPR in EU countries and GDPR barriers for the research involving identifiable individual-level data need to be addressed. GDPR interpretations in different countries and different institutions should be researched further and comparison should be made between those interpretations as a step towards univocal interpretation of the GDPR. Collaboration between the scientific community and regulators is needed to address the GDPR barriers for the research involving identifiable individual-level.

More research is needed to better understand the effects of GDPR on the secondary use of health data which can serve as a basis for future changes in the regulation.

## Conclusions

Our analysis showed that GDPR acts as both an enabler and a barrier for the secondary use of health data in Europe.

Lessons learned based on our analysis:

On one hand GDPR enables user`s rights over their data, increases patients` trust towards sharing their data, enables sharing of the anonymized statistics, increases transparency between partners and facilitates the introduction of new data analysis approaches which do not require a full data set to be shared which in turn minimizes data privacy risk. This represents a clear benefit to all the member states, researchers and citizens of European Union.

On the other hand, both the implementation and the interpretation of the GDPR varied a lot between EU member states (and within different institutions within the member states) which represents a clear barrier. It is important to ensure a univocal implementation of the GDPR in all the member states as well as a clear and univocal interpretation of the GDPR within all the member states and institutions within them. Since the implementation of the GDPR there are much more concerns about data protection which makes it more difficult to share data for scientific purposes. While GDPR in itself does not prohibit data sharing for scientific purposes, different interpretations and unclear understanding of it may cause difficulties. When it comes to dealing with identifiable and individual-level data, GDPR represents an issue with health data which is narrowly defined as it could be a way of identifying individuals and it makes health research on rare diseases more difficult as it is a field where the data is potentially identifiable. We see a need for deeper collaboration and discussion between the researchers in that field and the regulators to make research on rare events more feasible while at the same time complying with the potential privacy concerns. GDPR sometimes leads to a restricted access to data as the data providers are concerned about eventual violation of the data protection laws or they use GDPR as an excuse not to share the data, in our opinion a univocal interpretation of the GDPR might remove the restrictions for many of the data providers.

## Data Availability

The datasets used and/or analyzed during the current study are available from the corresponding author on reasonable request.

## Abbreviations

GDPR: General Data Protection Regulation
EU: European Union
HDN: Health Data Navigator
CORDIS: Community Research and Development Information Service
CIPH: Croatian Institute of Public Health
ICD: International Classification of Disease
GÖG: Austrian national public health institute (Gesundheit Österreich GmbH
